# Supplementary figures and images for: Combined Repair and Reconstruction of Coracoclavicular and Acromioclavicular Ligaments for Acute and Chronic AC Joint Dislocations: A Technical Note and Prospective Case Series
Source: J Clin Med. 2025 Mar 4;14(5):1730. doi: 10.3390/jcm14051730 (PMC11901272; doi:10.3390/jcm14051730)

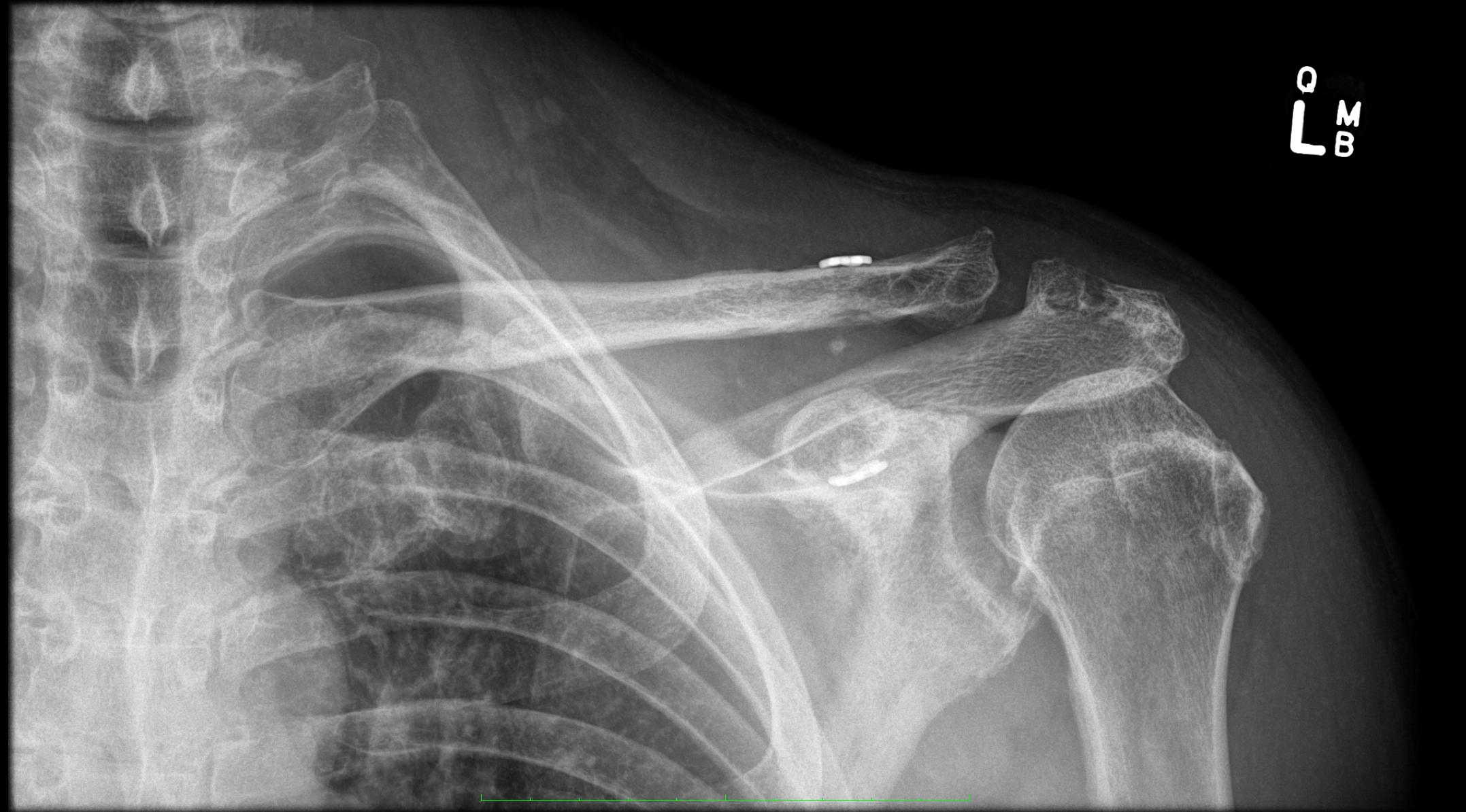

Supplement: Supplementary file 1 [file jcm-14-01730-s001.zip › Figure S1. Post-Operative X-Ray.png]

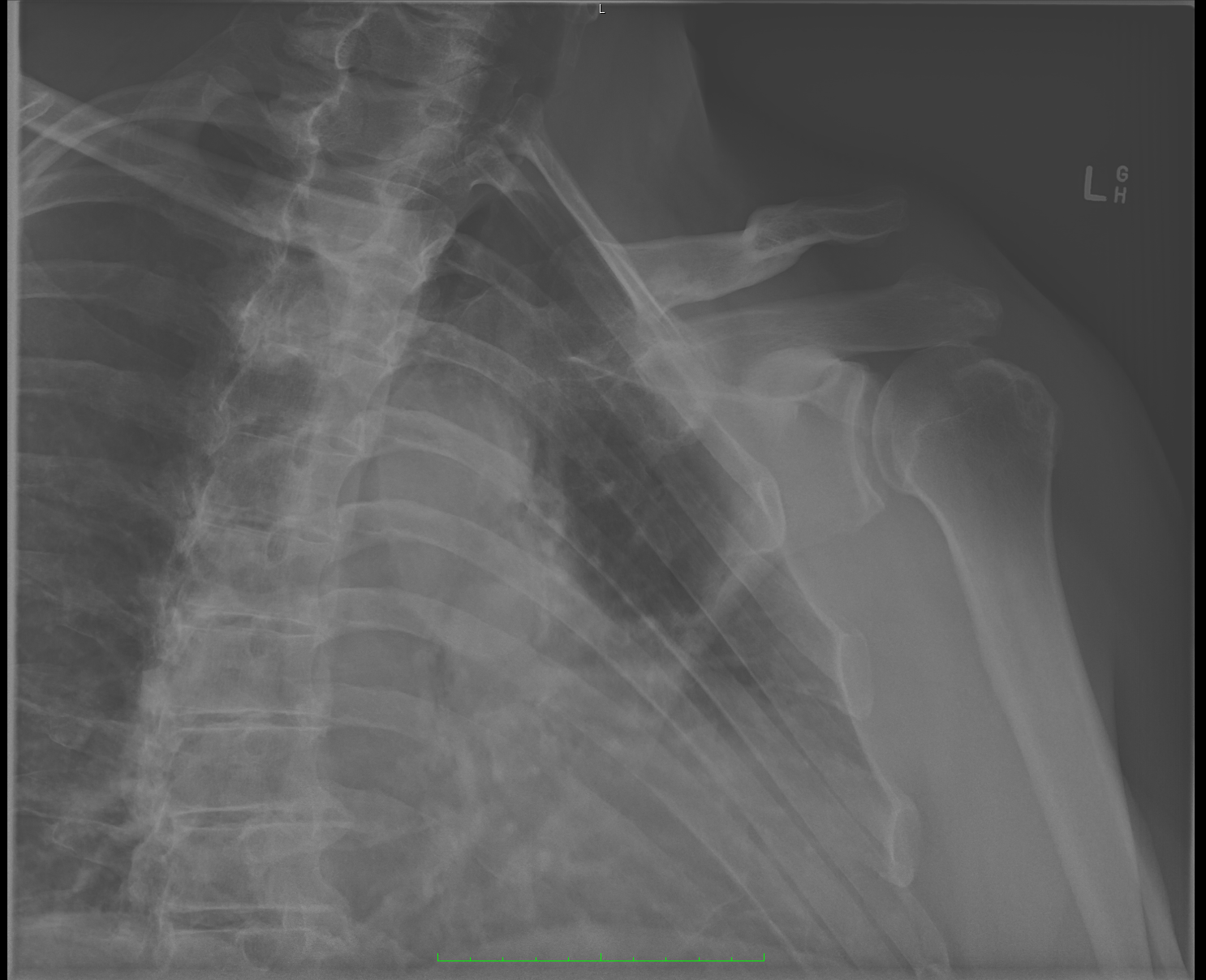

Supplement: Supplementary file 1 [file jcm-14-01730-s001.zip › Figure S2. Pre-Operative X-Ray.png]
